# Genome Assembly and Population Resequencing Reveal the Geographical Divergence of Shanmei (*Rubus corchorifolius*)

**DOI:** 10.1016/j.gpb.2022.05.003

**Published:** 2022-05-25

**Authors:** Yinqing Yang, Kang Zhang, Ya Xiao, Lingkui Zhang, Yile Huang, Xing Li, Shumin Chen, Yansong Peng, Shuhua Yang, Yongbo Liu, Feng Cheng

**Affiliations:** 1 Institute of Vegetables and Flowers, Chinese Academy of Agricultural Sciences, Key Laboratory of Biology and Genetic Improvement of Horticultural Crops, Ministry of Agriculture and Rural Affairs, Sino-Dutch Joint Laboratory of Horticultural Genomics, Beijing 100081, China; 2 Biotechnology Research Center, Xiangxi Academy of Agricultural Sciences, Jishou 416000, China; 3 Lushan Botanical Garden, Chinese Academy of Sciences, Jiujiang 332900, China; 4 State Environmental Protection Key Laboratory of Regional Eco-process and Function Assessment, Chinese Research Academy of Environmental Sciences, Beijing 100012, China

**Keywords:** *Rubus corchorifolius*, Genome assembly, Resequencing, Divergence, Genome evolution

## Abstract

***Rubus corchorifolius*** (Shanmei or mountain berry, 2*n* = 14) is widely distributed in China, and its fruits possess high nutritional and medicinal values. Here, we reported a high-quality chromosome-scale **genome assembly** of Shanmei, with contig size of 215.69 Mb and 26,696 genes. Genome comparison among Rosaceae species showed that Shanmei and Fupenzi (*Rubus chingii* Hu) were most closely related, followed by blackberry (*Rubus occidentalis*), and that environmental adaptation-related genes were expanded in the Shanmei genome. Further **resequencing** of 101 samples of Shanmei collected from four regions in the provinces of Yunnan, Hunan, Jiangxi, and Sichuan in China revealed that among these samples, the Hunan population of Shanmei possessed the highest diversity and represented the more ancestral population. Moreover, the Yunnan population underwent strong selection based on the nucleotide diversity, linkage disequilibrium, and historical effective population size analyses. Furthermore, genes from candidate genomic regions that showed strong **divergence** were significantly enriched in the flavonoid biosynthesis and plant hormone signal transduction pathways, indicating the genetic basis of adaptation of Shanmei to the local environment. The high-quality assembled genome and the variome dataset of Shanmei provide valuable resources for breeding applications and for elucidating the **genome evolution** and ecological adaptation of *Rubus* species.

## Introduction

*Rubus corchorifolius*, also named “Shanmei”, belongs to the Rosaceae family. Shanmei is described as an international “third-generation fruit” by the United Nations Food and Agriculture Organization due to its high economic and medicinal values [1]. Shanmei fruits are popular due to its unique flavor and nutrients, including high amounts of anthocyanins, superoxide dismutase, vitamin C, and essential amino acids [2]. Shanmei fruits are processed into food products such as jam, juice, wine, and ice cream, which are becoming increasingly popular among consumers [3]. A previous study found that the terpenoids synthesized in Shanmei can suppress the development of cancer cells by inducing tumor cell differentiation and apoptosis [4]. Despite its growing economic importance and medicinal value, the genetic and genomic exploitation of Shanmei is very limited, which hinders its practical utilization.

**Figure 1:**
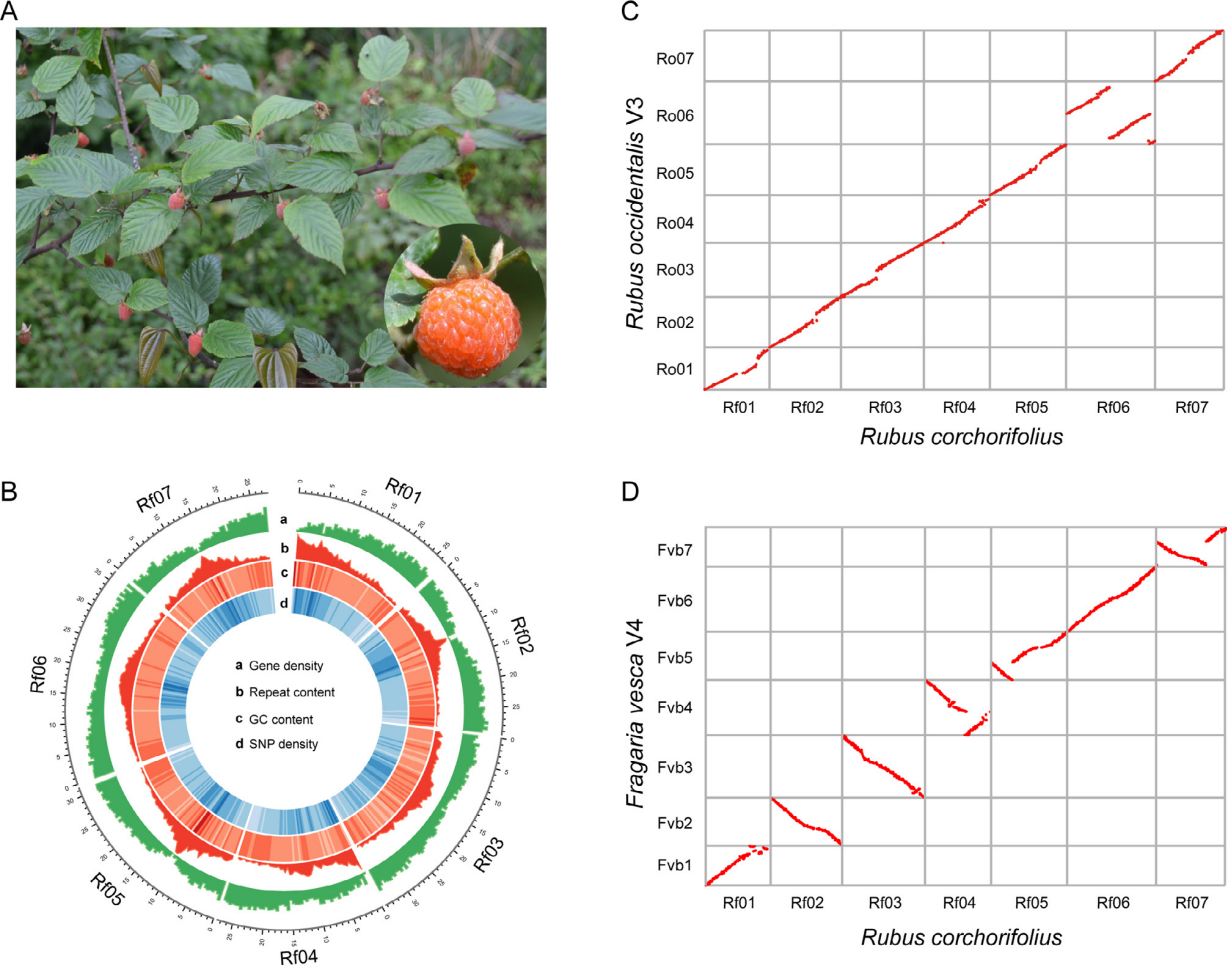
**Assembly and characterization of the Shanmei genome** **A.** Shanmei plant with a close-up view of its fruit. **B.** The landscape of the Shanmei genome. The chromosome units are in 1 Mb. **C.** Genomic synteny between Shanmei (*Rubus corchorifolius*) and blackberry (*Rubus occidentalis*). **D.** Genomic synteny between Shanmei (*Rubus corchorifolius*) and strawberry (*Fragaria vesca*). SNP, single nucleotide polymorphism.

Shanmei is a broadly distributed species that has not been subjected to extensive domestication or cultivation selection as of yet. It is one of the approximately 750 species in the *Rubus* genus. *Rubus* is globally distributed and is well-adapted to a broad range of environments, from extremely cold climates near the polar regions and the high plateau of the Himalayas, to temperate and hot climates in tropical areas, such as the highlands of the Andes [5]. *Rubus* species constitute important components of the ground layer of hillsides, valleys, and large forest canopy gaps, providing a host of ecological benefits (including soil stabilization and reduced soil nutrient loss), as well as food for wildlife [6]. At present, only a few species in the *Rubus* genus, including *Rubus occidentalis* (blackberry), *Rubus caesius* (dewberry), and *Rubus arcticus* (arctic raspberry), have been domesticated and utilized in breeding programs [7]. Some of them, such as the blackberry, have been developed as important crops with great economic values [8]. Shanmei is a deciduous erect shrub with prickly stems and leaves that have pointed lobes near the base of the blade (Figure 1A). It is one of the Asian representative species of *Rubus*. Shanmei is native to China [9] and is mainly distributed in South China (https://www.cvh.ac.cn). Shanmei populations from different geographical regions of South China exhibit great phenotypic diversity, including in characters associated with environmental adaptation and population size, and in various fruit traits. The wide distribution and high diversity of Shanmei populations suggest great potential for their exploitation and agricultural utilization [6]. Additionally, South China has been proposed as a potential location for the spread of Shanmei, and Shanmei populations from different geographical regions are under extensive natural selection. However, the origin and evolutionary features of Shanmei populations have not been elucidated.

Genome sequencing provides a direct and efficient means of studying the origin and population evolutionary features of species of interest, as well as exploring the genes associated with traits [10]. Thus far, more than 10 high-quality genomes of Rosaceae species have been released, including *Pyrus communis* (pear) [11], *Fragaria vesca* (strawberry) [12], and *Rosa chinensis* (rose) [13]. Among them, only two sequenced genomes are from *Rubus*, namely, blackberry [8] and *Rubus chingii* Hu (Fupenzi) [14]. Research using genome sequences of blackberry showed that duplication of the gene encoding chalcone synthase, the first committed enzyme in flavonoid biosynthesis, was positively correlated with trait domestication in blackberry [8]. For Fupenzi, analysis based on genome sequencing revealed that there was a tandem gene cluster in chromosome 2 that regulated the biosynthetic pathway of hydrolyzable tannins [14]. Genome resequencing (variome) shows advantages in investigations of population divergence and genes related to important traits, which can facilitate the development of molecular breeding markers for these traits. For example, genome resequencing revealed the domestication history and the genetic mechanisms underlying important agronomic traits in fruit, including flavor, scent, nutritional value, flower color, and flowering time, in apple [15], citrus [16], grape [17], and others [18], [19], [20]. Additionally, using chloroplast genomic data, researchers have shown that Shanmei is phylogenetically located in the Rubeae clade of the Rosaceae family and is most closely related to *Rubus rufus* [21]. However, as there are no available genomic and population variome resources for Shanmei, the genomic evolution, geographical origin, and population structure features, as well as the genes related to the adaptation and selection of this potentially important species, are largely unknown.

In this study, we generated the first chromosome-scale assembly of the Shanmei genome. Comparative genomics analysis of the Shanmei genome with the genomes of 10 Rosaceae species revealed the expanded gene families that allowed Shanmei to occupy its special ecological niche in various wild regions. Guided by the density distribution of Shanmei in China, we collected samples from four representative regions and performed resequencing and population genomic analysis. We found that the Hunan population, which is located in the middle region of South China, was the ancestral group of Shanmei, while genes associated with flavonoid- and phytohormone-related pathways were under strong selection in the Yunnan group located at the high-altitude regions in Southeast China. These findings based on the high-quality assembled genome and the population variome dataset of Shanmei not only provide insights into its evolution, geographical divergence, and adaptation but also provide a foundation for its further agricultural utilization as a fast-growing and economical horticultural crop.

## Results

### Pseudo-chromosome construction of the Shanmei genome

**Supplementary Figure S1:**
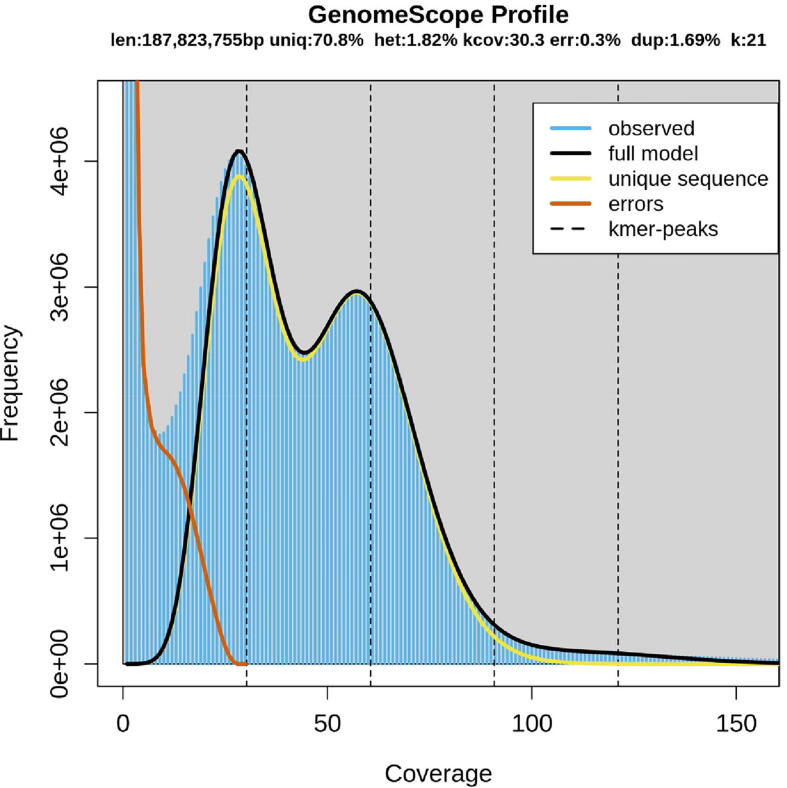
**Figure S1**  **The genome size of Shanmei estimated by GenomeScope** len, genome length; uniq, percentage of unique *k-mer*s; het, heterozygosity rate; kcov, read coverage; err, read error rate; dup, percentage of repetitive content; k, length of *k-mer*.

**Supplementary Figure S2:**
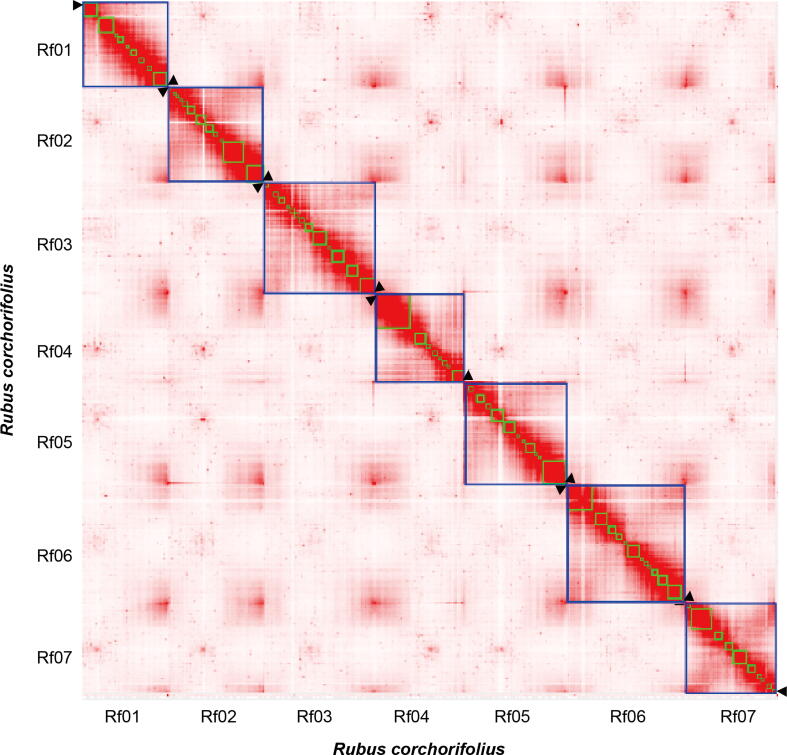
**Figure S2**  **Whole-genome Hi-C contacts of Shanmei** The black triangles represent the positions of telomere sequences in the seven chromosomes of Shanmei. Hi-C, high-throughput chromosome conformation capture.

We sequenced and assembled the genome of Shanmei using combined sequencing datasets from Oxford Nanopore Technologies (ONT), Illumina HiSeq, and high-throughput chromosome conformation capture (Hi-C). The genome was estimated to be 187.82 Mb in size with a heterozygosity ratio of 1.82% based on 21-mer counting, showing that it is highly heterozygous (Figure S1). A total of 36.87 Gb (∼ 180×) ONT reads were generated and assembled into 221 contigs (Table S1). The size of the assembly was 330.25 Mb, with a contig N50 of 2.49 Mb. It was speculated that the larger size of the assembly compared to the estimate was caused by the introduction of the heterozygous contigs, considering the high heterozygosity ratio. Therefore, redundant contigs were then identified and filtered out using Purge Haplotigs (version 1.2.3) [22]. After that, only 120 contigs (215.69 Mb) were retained for further analysis (Table 1). A total of 43.56 Gb (∼ 220×) Hi-C data were further used to link the contigs into scaffolds. Consequently, 10 scaffolds were obtained with an N50 of 29.50 Mb. The seven largest scaffolds comprised 117 contigs, which accounted for 99.35% (214.29 Mb) of the assembled genome and corresponded to the seven pseudo-chromosomes of Shanmei (Figure 1B, Figure S2; Table S2). Furthermore, the telomere sequences were identified at the ends of the seven chromosomes (Figure S2), which supported the relative completeness of the assembled genome of Shanmei. Additionally, Benchmarking Universal Single-Copy Orthologs (BUSCO) analysis showed that 94.70% of the BUSCO genes were successfully identified in the Shanmei genome.

**Table 1:** **Assembly and annotation statistics of the Shanmei genome**

**Type**	**Contig**		**Scaffold**	
	**Size (Mb)**	**Number**	**Size (Mb)**	**Number**
Maximum	11.08	1	36.68	1
N50	3.34	21	29.50	4
N90	0.78	80	27.00	6
Total	215.69	120	215.74	10
Chromosomes	/	/	214.29 (99.35%)	
Protein-coding genes	/	/	/	26,696
Repetitive elements	/	/	77.33 (35.85%)	/

We employed an integrated pipeline to annotate the genome by combining *de novo* prediction, homology search, and RNA-seq data alignment (see Materials and methods). A total of 26,696 protein-coding genes were predicted in the Shanmei genome (Table S3). The high gene prediction quality was supported by the fact that 1976 (93.1%) of the BUSCO genes were found in the Shanmei gene set. In addition, repeat annotation revealed that approximately 35.85% (77.33 Mb) of the genome was composed of repetitive elements, which is comparable to that of Fupenzi [14]. The predominant type of transposable elements was long terminal repeat retrotransposons, accounting for 11.26% of the genome (Table S4).

### Expanded gene families were involved in flavonoid biosynthesis and stress resistance

**Figure 2:**
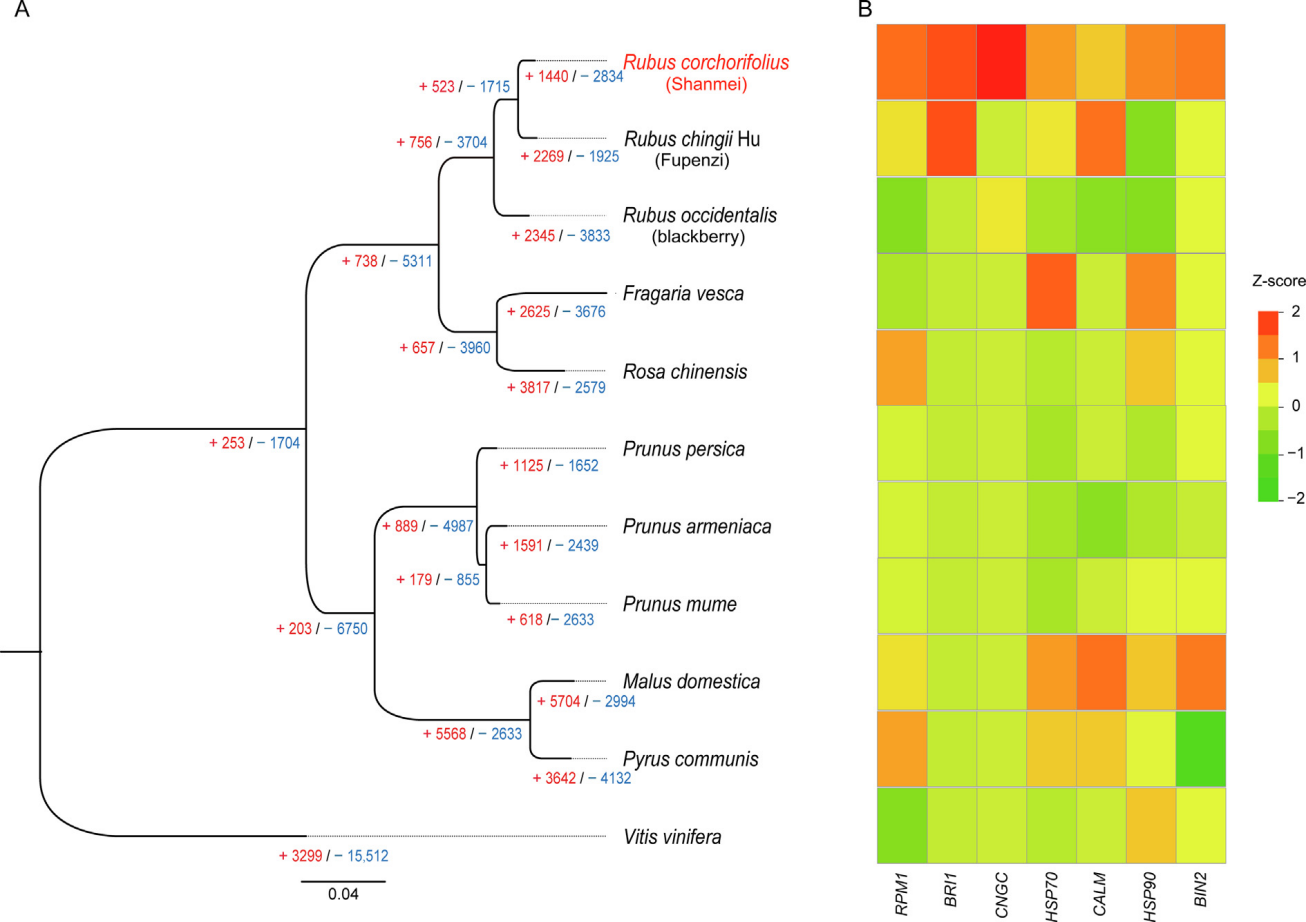
**Phylogenetic position and gene family expansion of Shanmei** **A.** The phylogenetic tree of Shanmei and nine other Rosaceae species built based on 932 single-copy genes, with *Vitis vinifera* as the outgroup. The inferred expansion (red numbers) and contraction (blue numbers) of gene families in different genomes are indicated. **B.** Copy number variation of the gene families associated with environmental adaptation.

**Supplementary Figure S3:**
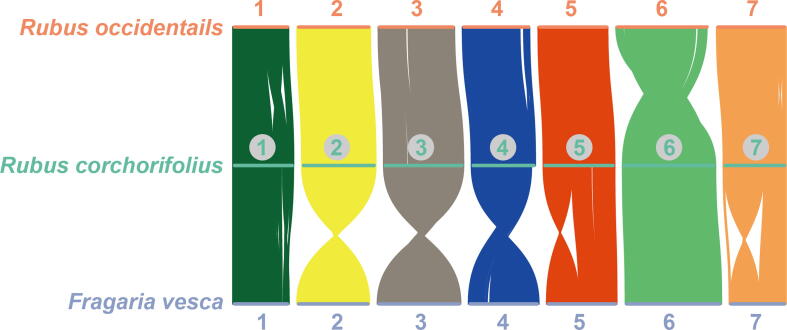
**Figure S3**  **Genome synteny analysis of Shanmei, blackberry, and strawberry by MCscanX** The numbers indicate the chromosome order. The line represents a one-to-one correspondence of homologous regions between genomes of Shanmei and blackberry or strawberry.

**Supplementary Figure S4:**
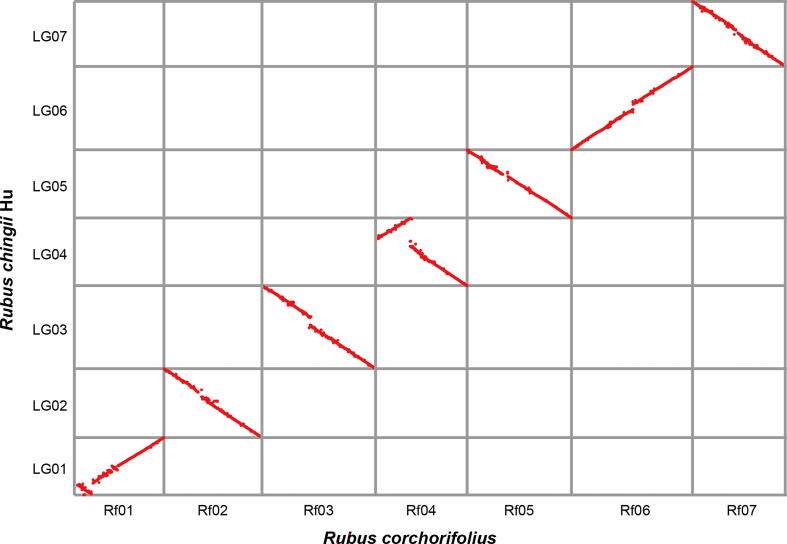
**Figure S4**  **Genomic synteny between Shanmei and Fupenzi** *Rubus chingii* Hu, Fupenzi.

**Supplementary Figure S5:**
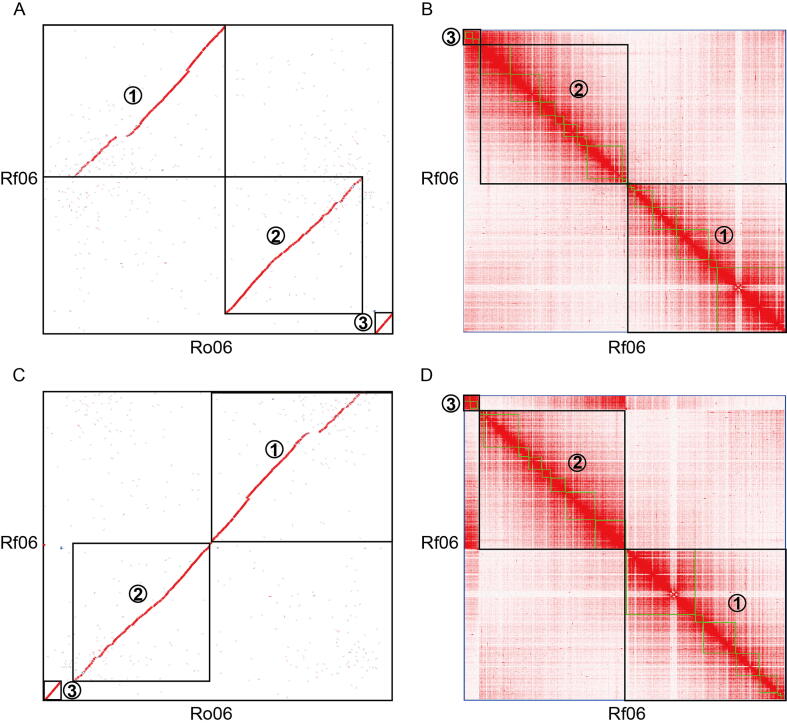
**Figure S5**  **Verification of the segmental translocation in chromosome 6 between Shanmei and blackberry using the information of Hi-C contacts** **A.** The synteny of chromosome 6 between Shanmei and blackberry. The X-axis denotes the chromosome 6 of blackberry (Ro06). The Y-axis denotes the chromosome 6 of Shanmei (Rf06). **B.** The Hi-C heatmap of Shanmei Rf06. **C.** The synteny of chromosome 6 between Shanmei and blackberry after the re-ordering of Shanmei Rf06 following that of blackberry Ro06. **D.** The Hi-C heatmap of Shanmei Rf06 after re-ordering. There are obvious incorrect Hi-C contacts in the re-ordered Rf06 of Shanmei.

**Supplementary Figure S6:**
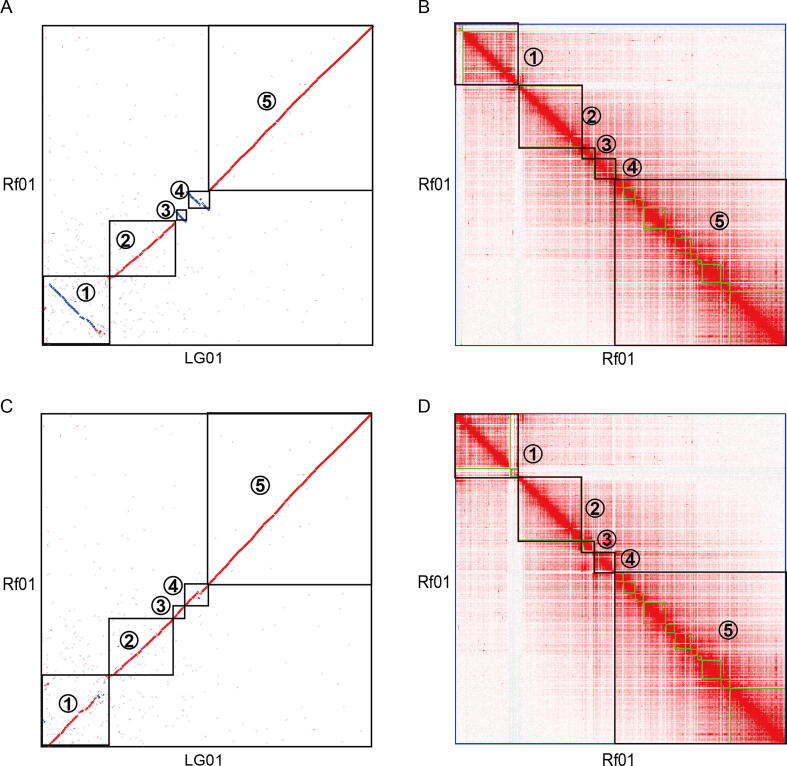
**Figure S6**  **Verification of the inversions in chromosome 1 between Shanmei and Fupenzi using the information of Hi-C contacts** **A.** The synteny of chromosome 1 between Shanmei and Fupenzi. The X-axis denotes chromosome 1 of Fupenzi (LG01). The Y-axis denotes the chromosome 1 of Shanmei (Rf01). **B.** The Hi-C heatmap of Shanmei Rf01. **C.** The synteny of chromosome 1 between Shanmei and Fupenzi after the re-ordering of Shanmei Rf01 following that of Fupenzi LG01. **D.** The Hi-C heatmap of Shanmei Rf01 after re-ordering.

**Supplementary Figure S7:**
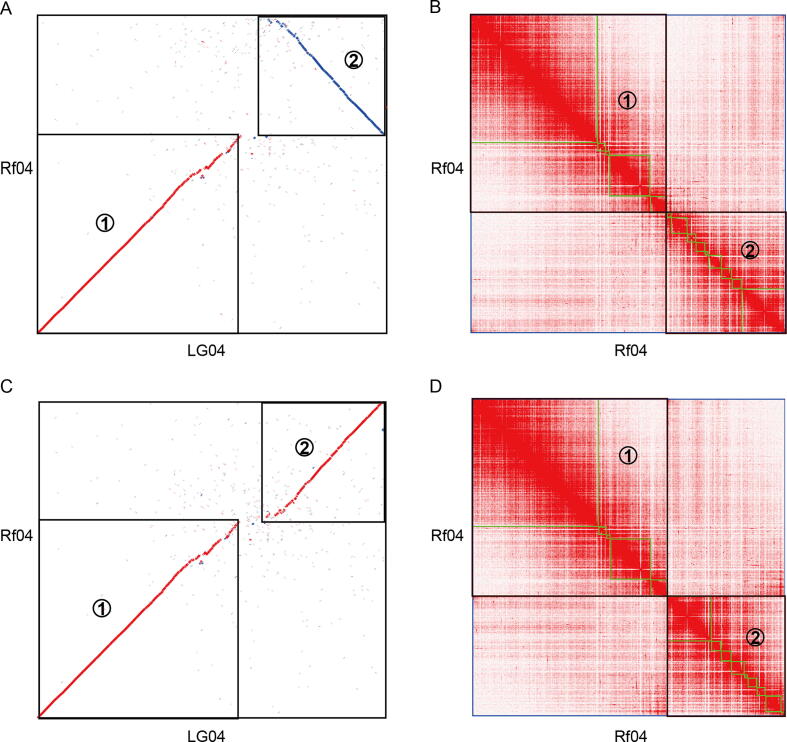
**Figure S7**  **Verification of the inversion in chromosome 4 between Shanmei and Fupenzi using information of Hi-C contacts** **A.** The synteny of chromosome 4 between Shanmei and Fupenzi. The X-axis denotes chromosome 4 of Fupenzi (LG04). The Y-axis denotes chromosome 4 of Shanmei (Rf04). **B.** The Hi-C heatmap of Shanmei Rf04. **C.** The synteny of chromosome 4 between Shanmei and Fupenzi after the re-ordering of Shanmei Rf04 following that of Fupenzi LG04. **D.** The Hi-C heatmap of Shanmei Rf04 after re-ordering.

Rosaceae is an economically important family composed of 2800 species among 95 genera, including the specialty fruit crops apple, almond, and blackberry. In order to infer the phylogenetic position of Shanmei in Rosaceae, we obtained 932 single-copy genes and constructed a phylogenetic tree for 10 Rosaceae species with grape as the outgroup (Figure 2A). The results showed that Shanmei and Fupenzi were most closely related. Each genomic region in Shanmei was found to be orthologous to a single genomic region in Fupenzi, blackberry, and strawberry based on genomic synteny analysis, suggesting that no lineage-specific genome duplication occurred in Shanmei after the common *γ* hexaploidization event shared by eudicots [8] (Figure 1C and D, Figures S3 and S4). In addition, a large translocation on chromosome 6 between Shanmei and blackberry was identified (Figure 1C). To verify the accuracy of the assembly, we re-adjusted the scaffolding orders of the contigs in chromosome 6 to follow those in blackberry and found that the resultant Hi-C heatmap exhibited clear mis-connections (Figure S5), suggesting an authentic translocation between Shanmei and blackberry, which may be associated with the divergence of the two species. Meanwhile, four smaller inversions on chromosomes 1 and 4 were found between Shanmei and Fupenzi (Figure S4). These inversions were also verified using Hi-C heatmaps (Figures S6 and S7).

**Supplementary Figure S8:**
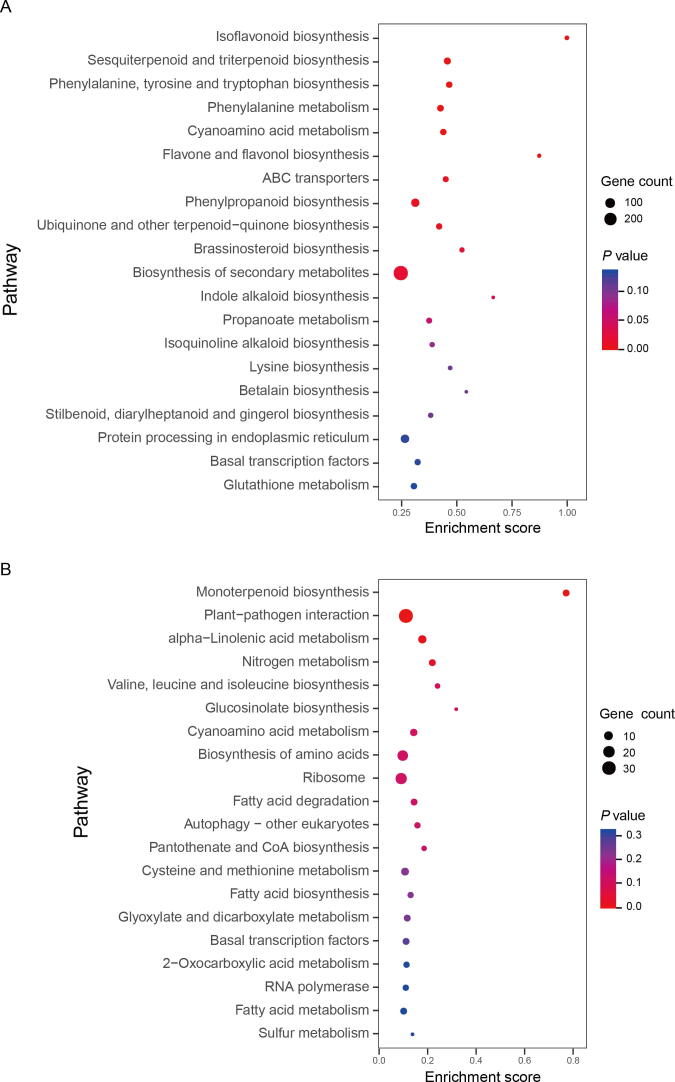
**Figure S8**  **KEGG enrichment analysis of gene sets in Shanmei** **A.** KEGG enrichment for expanded genes. **B.** KEGG enrichment for contracted genes. KEGG, Kyoto Encyclopedia of Genes and Genomes.

Subsequently, we determined the expansion and contraction of orthologous gene families using CAFÉ (version 4.2.1) [23]. We found that a total of 1440 and 2834 gene families underwent expansion and contraction, respectively. Kyoto Encyclopedia of Genes and Genomes (KEGG) enrichment analyses revealed that the expanded gene families mainly participated in phenylalanine metabolism, flavonoid biosynthesis, brassinosteroid biosynthesis, and biosynthesis of secondary metabolites (corrected *P* < 0.05; Figure S8A; Table S5). In contrast, the contracted gene families were mainly associated with monoterpenoid biosynthesis, alpha-linolenic acid metabolism, and nitrogen metabolism (corrected *P* < 0.05; Figure S8B; Table S6). Furthermore, some significantly expanded genes were closely related to stress resistance, such as *HSP90*, *HSP70*, *BRI1*, *BIN2*, and *RPM1* (Figure 2B; Table S7). Multiple studies have reported that the expanded families observed here contribute to abiotic and biotic stress tolerance in different plants. For example, the overexpression of *OsHSP90* can enhance cell viability and heat tolerance in rice under heat stress [24]; *BRI1*, encoding a signal receptor in the brassinosteroid signal transduction pathway, plays an important role in plant development and disease resistance [25]; and *RPM1* is a resistance gene that improves the resistance to root-knot nematodes in wild myrobalan plum [26]. In summary, the expansion of these genes may contribute to the environmental adaptability of Shanmei in the wild.

### Genomic variation and morphology

**Supplementary Figure S9:**
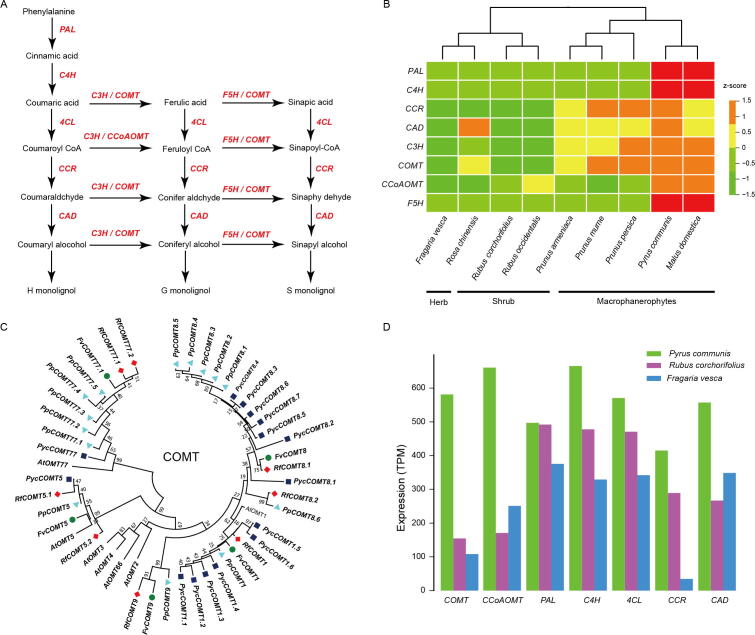
**Figure S9**  **Variations in copy number and expression of key genes involved in lignin biosynthesis in Rosaceae** **A.** The genes reported in the biosynthesis pathway of lignin. **B.** Heatmap of the copy number of key genes for lignin biosynthesis in Rosaceae. **C.** Phylogenetic tree of the *COMT* gene family. The numbers are the bootstrap values. The light blue triangles, blue boxs, green circles, and red rhombuses represent the genes of peach, pear, strawberry, and Shanmei, respectively. **D.** The gene expression level measured by mRNA-seq data of the stem organ from three representative species of macrophanerophytes, shrub, and herb. Rf, Shanmei; Fv, strawberry; Pp, peach; Pyc, pear; At, *Arabidopsis*; TPM: transcripts per million mapped reads; *PAL*, phenylalanine ammonialyase; *C4H*, cinnamate 4-hydroxylase; *C3H*, coumarate 3-hydroxylase; *COMT*, caffeic acid O-methyltransferase; *F5H*, ferulate 5-hydroxylase; *4CL*, CoA ligase; *CCoAOMT*, caffeoyl-CoA O-methyltransferase; *CCR*, cinnamoyl-CoA reductase; *CAD*, cinnamyl alcohol dehydrogenase.

Shanmei is a low shrub, which is typical of the *Rubus* genus. Lignin is an important factor associated with plant height differences [27]. We identified the key genes for lignin biosynthesis in Shanmei based on their homologous genes reported in *Arabidopsis thaliana.* We found that the gene copy numbers of *CAD* (*P* = 0.036) and *COMT* (*P* = 0.047) increased significantly in trees (Figure S9; Table S8). There are 9 copies of *CAD* in Shanmei and 12 in strawberry, compared to 24 and 18 in trees of pear and peach, respectively. A previous study has found that the decreased expression of *CAD* led to sterility and dwarfing in *A. thaliana* [28]. The increased copy number of *CAD* in trees may contribute to their lignin biosynthesis activity. Meanwhile, the copy number of *COMT* in shrubs (8 in Shanmei) was higher than that in herbs (5 in strawberry), but both were lower than those in trees (15 in pear and 14 in peach) (Figure S9C). COMT is one of the important enzymes controlling lignin monomer production in plant cell wall biosynthesis, and decreased expression of *COMT* resulted in a decreased lignin content [29]. Furthermore, we compared the expression of genes related to lignin biosynthesis in three representative species, *i.e.*, strawberry, Shanmei, and pear, and found that the expression levels of lignin biosynthesis-related genes showed a positive association with the heights of the species that were compared (Figure S9D), which further supported the dosage effect of these lignin biosynthesis-related genes in Rosaceae.

**Supplementary Figure S10:**
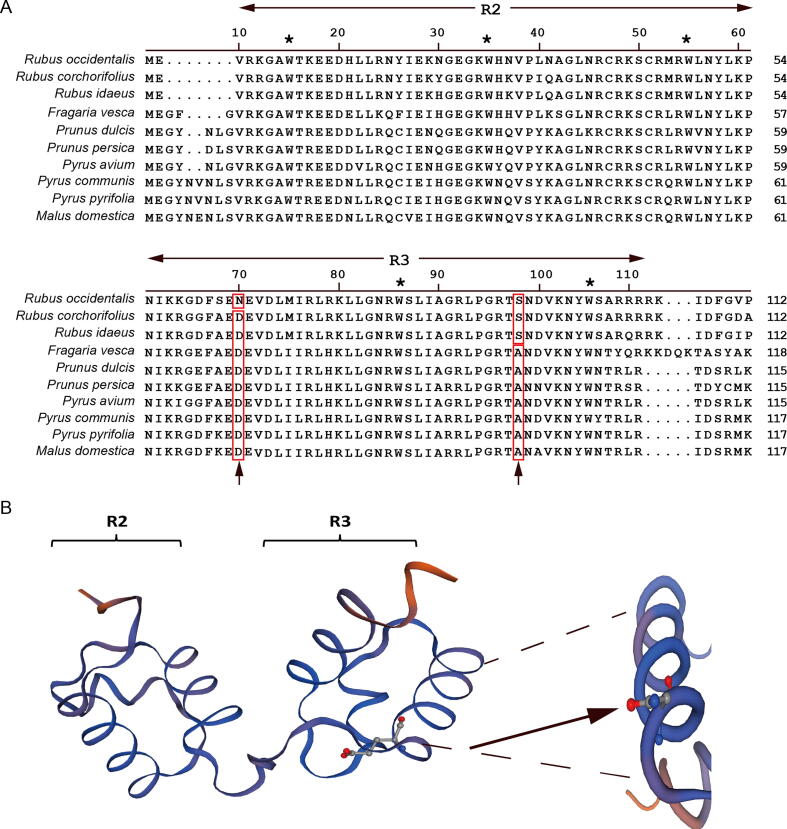
**Figure S10**  **Characterization of MYB10 in Rosaceae species** **A.** Protein sequence alignment of the MYB10 transcription factors, showing only part of the R2 and R3 domains. Conserved tryptophan residues in the R2 and R3 domains are marked with asterisks (*). The characteristic amino acids in the dicot anthocyanin-promoting MYB transcription factors are highlighted in red boxes. **B.** The RuMYB10 protein 3D structure. The arrow points at the asparagine (N). *Rubus idaeus*, red raspberry; *Prunus dulcis*, almond; *Prunus persica*, peach; *Pyrus avium*, sweet cherry; *Pyrus communis*, pear; *Pyrus pyrifolia*, sand pear; *Malus domestica*, apple.

Anthocyanins are abundant in Shanmei and have essential functions in stress resistance and fruit coloring. We identified the key genes for anthocyanin biosynthesis in the Shanmei genome based on the anthocyanin-related gene pathways reported in *Arabidopsis* and blackberry [30], [31] (see Materials and methods; Table S9). Among them, *MYB10* is the main regulator in anthocyanin biosynthesis. By comparing the functional domain of MYB10 among 10 species of Rosaceae, we identified two conserved motifs (R2 and R3 as shown in Figure S10A) in MYB10, and found that alanine (A) in the R3 motif was substituted by serine (S) in Shanmei, as well as in red raspberry and blackberry [31]. In addition, we found a novel substitution only in blackberry, in which aspartic acid (D, acidic amino acid) located in the R3 motif was replaced by asparagine (N, neutral amino acid) (Figure S10B).

### Population structure of Shanmei

**Figure 3:**
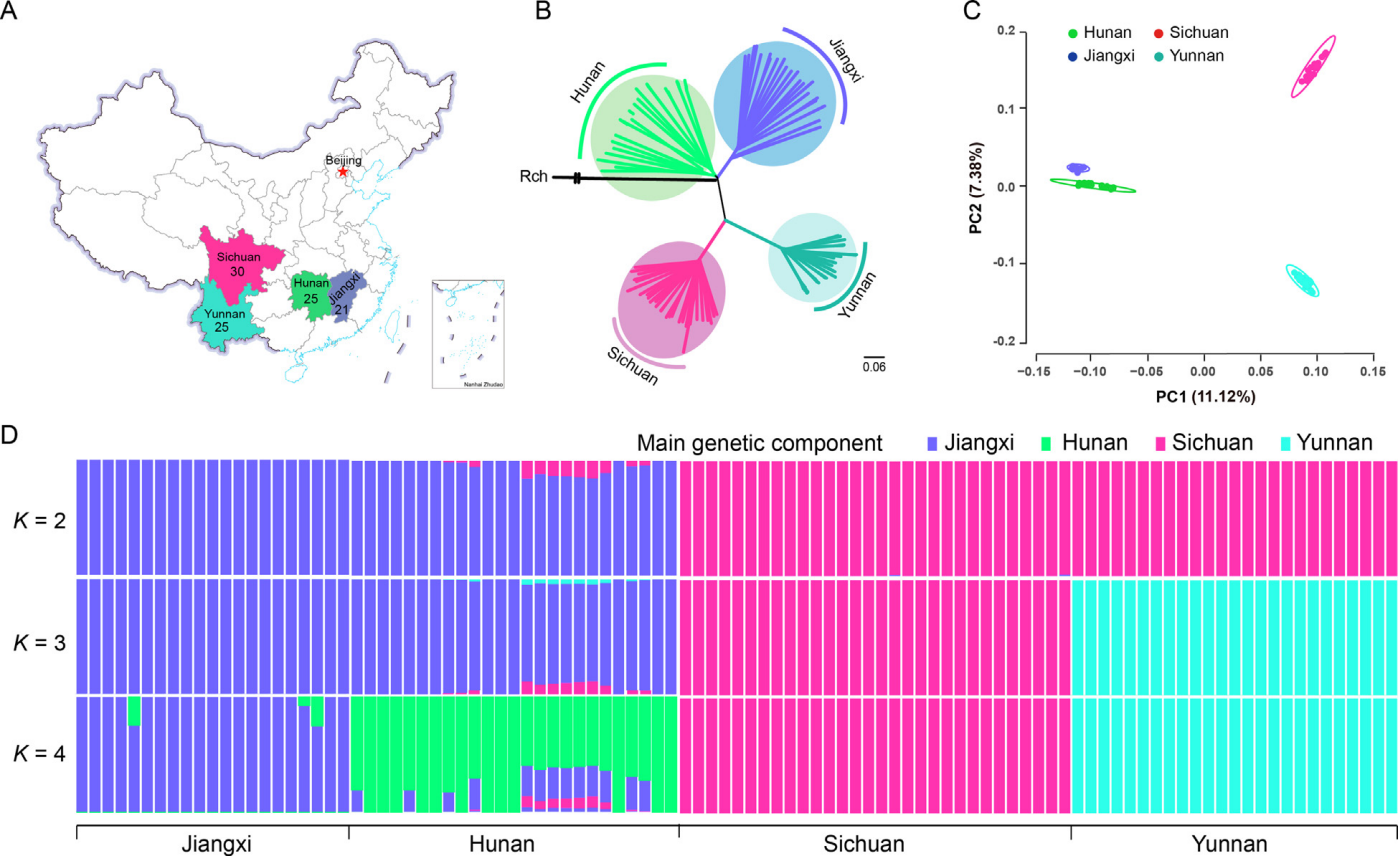
**Population structure of Shanmei** **A.** The geographic locations sampled in this study. The number denotes the number of samples collected in the corresponding region. **B.** Best ML tree showing the phylogenetic relationships of the 101 Shanmei samples. The genome of Fupenzi (Rch) was used as the outgroup. **C.** PCA of the Shanmei populations. **D.** Genetic admixture of the Shanmei samples. The length of each colored segment represents the proportion of genetic components in each sample (*K* = 2–4). Blue, green, pink, and cyan represent the main genetic components of Jiangxi, Hunan, Sichuan, and Yunnan Shanmei groups, respectively. ML, maximum likelihood; PCA, principal component analysis; PC, principal component.

To elucidate the population structure of Shanmei, we collected 101 samples from the provinces of Jiangxi (*n* = 21), Hunan (*n* = 25), Yunnan (*n* = 25), and Sichuan (*n* =30) in South China (see Materials and methods), corresponding to the main distribution area of Shanmei (Figure 3A). We resequenced these samples at an average depth of 35× coverage (Table S10). The mapping rate was 92.61% on average (Table S10). Single nucleotide polymorphisms (SNPs) were identified using Genome Analysis Toolkit (GATK) [32]. After filtering, a total of 758,978 SNPs were retained for further analysis. The SNPs were evenly distributed across the seven chromosomes (Figure 1B; Table S11), and 18.98% and 10.56% of the SNPs were located in gene-proximal (2-kb upstream or downstream of a coding sequence) and in coding regions, respectively. Moreover, a total of 38,468 (5.07%) SNPs resulted in non-synonymous sequence changes, among which 837 (0.11%) SNPs disrupted the coding sequence (premature stop codon).

**Supplementary Figure S11:**
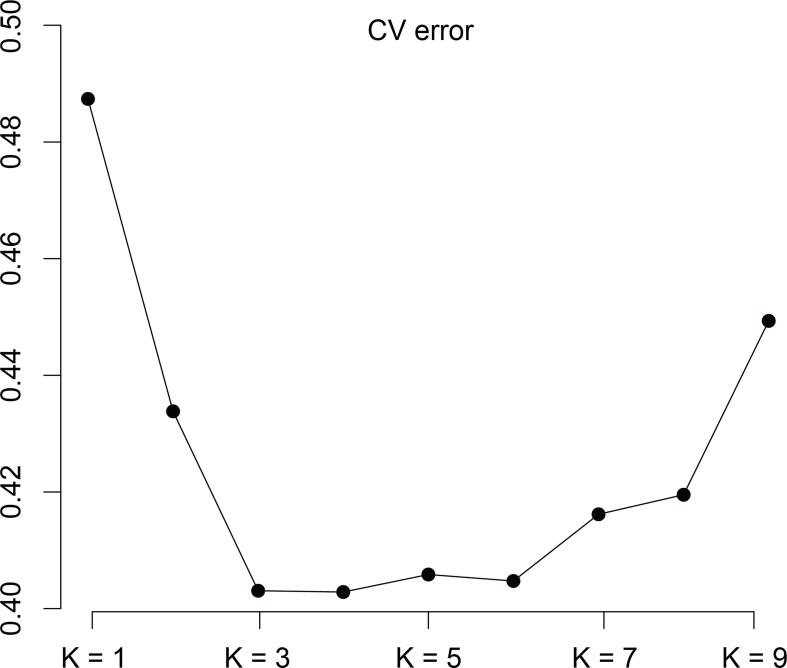
**Figure S11**  **Standard error estimation of Shanmei population admixture analysis** CV, cross-validation.

To further explore the phylogenetic relationships of the 101 samples, we constructed a phylogenetic tree using the maximum likelihood (ML) method and found that the accessions were clustered into four clades, which exactly corresponded to the four geographical regions (Figure 3B). Principal component analysis (PCA) also revealed four clusters, which was consistent with the phylogenetic result (Figure 3C). We found that the Jiangxi and Hunan groups remained closely associated. The clustering results were further confirmed by the genetic structure analysis (Figure 3D, Figure S11). When *K* = 4, the same four groups were obtained, indicating the distinct divergence among populations from different geographical regions. Furthermore, the phylogenetic tree with Fupenzi as the outgroup aslo indicated that the Hunan group was the ancestral group of Shanmei (Figure 3B). Overall, these results imply that the Hunan group is more diverse than the other groups of Shanmei.

### Flavonoid and phytohormone pathways contributed to the adaptation of Shanmei

**Figure 4:**
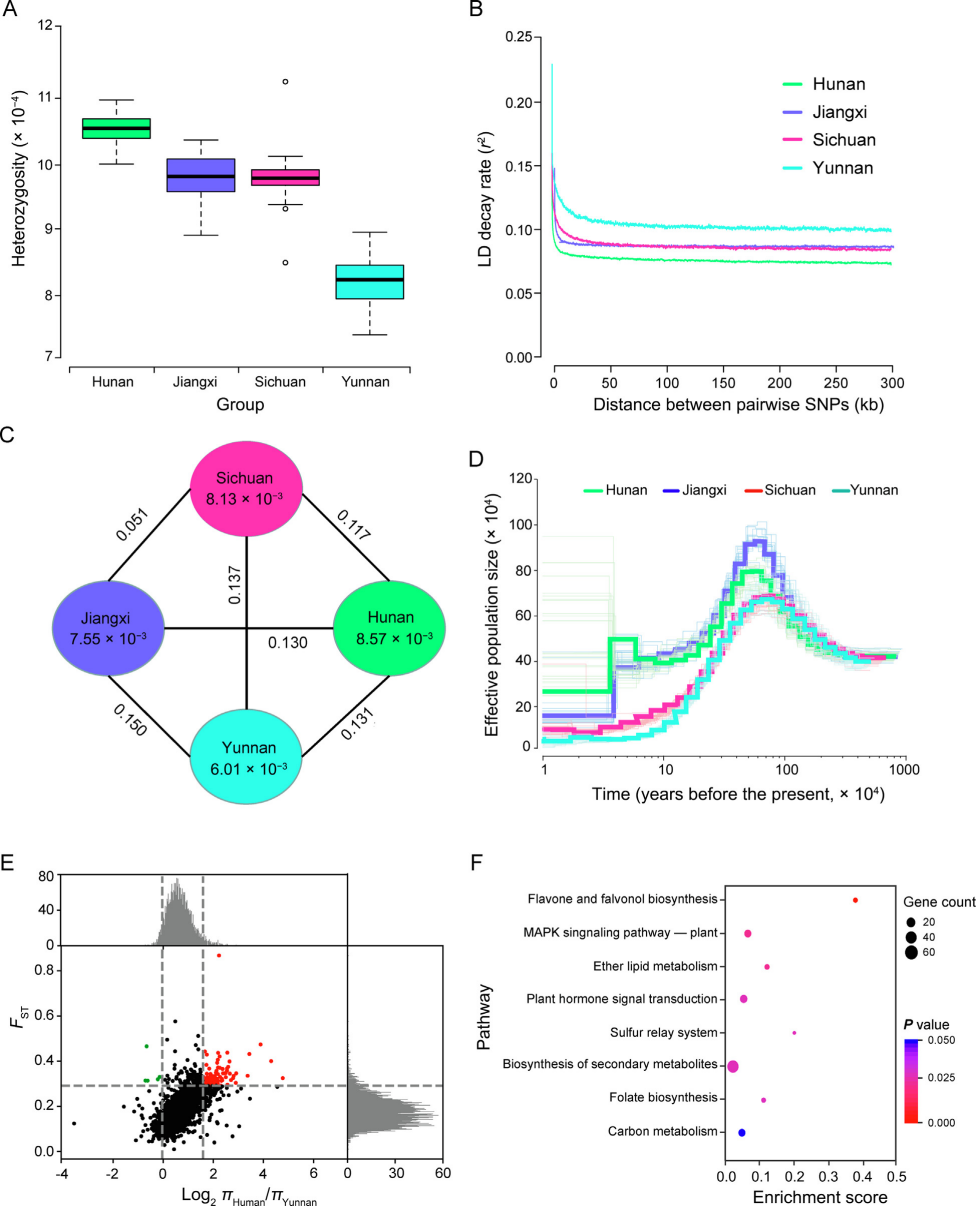
**Nucleotide diversity and population divergence of the 101 Shanmei samples** **A.** Genomic heterozygosity of the Hunan, Jiangxi, Sichuan, and Yunnan Shanmei groups. **B.** Decay of LD in four groups of Shanmei. **C.***π* and *F*_ST_ among the four Shanmei groups. Values between pairs indicate *F*_ST_, and values in circles represent *π* for corresponding group. **D.** Historical effective population size of four Shanmei groups. **E.** Distribution of *F*_ST_ and *π* ratio (log_2_*π*_Human_/*π*_Yunnan_) between the Hunan and Yunnan groups. *F*_ST_ and *π* values were calculated across the Shanmei genome using a 50-kb sliding window. **F.** Functional enrichment of genes located at genomic regions under selection in the Yunnan group. LD, linkage disequilibrium; *π*, nucleotide diversity; *F*_ST_, population divergence.

We further investigated the population-level heterozygosity in the Shanmei populations based on the results of the phylogenetic and population structure analyses. We found that the Yunnan group had a lower level of heterozygosity than the Jiangxi, Sichuan, and Hunan groups (Figure 4A; Table S12). Consistently, the decay rate of linkage disequilibrium (LD, indicated by *r*^2^) was highest in the Yunnan group followed by the Sichuan, Jiangxi, and Hunan groups (Figure 4B). We then calculated the nucleotide diversities (*π*) for the four groups. The Yunnan group had the lowest nucleotide diversity (*π* = 6.01 × 10^−3^) compared to the Sichuan (*π* = 8.13 × 10^−3^), Hunan (*π* = 8.57 × 10^−3^), and Jiangxi (*π* = 7.55 × 10^−3^) groups (Figure 4C). In addition, the historical effective population size analysis showed that the population size of Yunnan decreased significantly in the recent period compared to that of the other groups (Figure 4D). These results suggest that the Yunnan group, which is distributed in the high-altitude region, underwent the greatest selection pressure among the four groups.

To reveal the genetic basis of natural selection in the Yunnan group, the Hunan, Jiangxi, and Sichuan groups were used as controls to determine the candidate genomic regions under selection through genome scanning with a 50-kb sliding window. We found 97 regions that displayed increased levels of differentiation between the Yunnan group and Hunan group and a significant reduction in nucleotide diversity in the Yunnan group [fixation statistic (*F*_ST_) > 0.29; log_2_ *π*__Human__/*π*__Yunnan__ > 1.59; both exceeding the top 5% threshold] (Figure 4E; Table S13). Similarly, a total of 94 regions between the Jiangxi group and Yunnan group (Table S14), as well as 57 regions between the Sichuan group and Yunnan group (Table S15), were identified. In total, we identified 749, 679, and 435 genes in these candidate regions through the Hunan *vs.* Yunnan, Jiangxi *vs.* Yunnan, and Sichuan *vs.* Yunnan comparisons, respectively.

**Figure 5:**
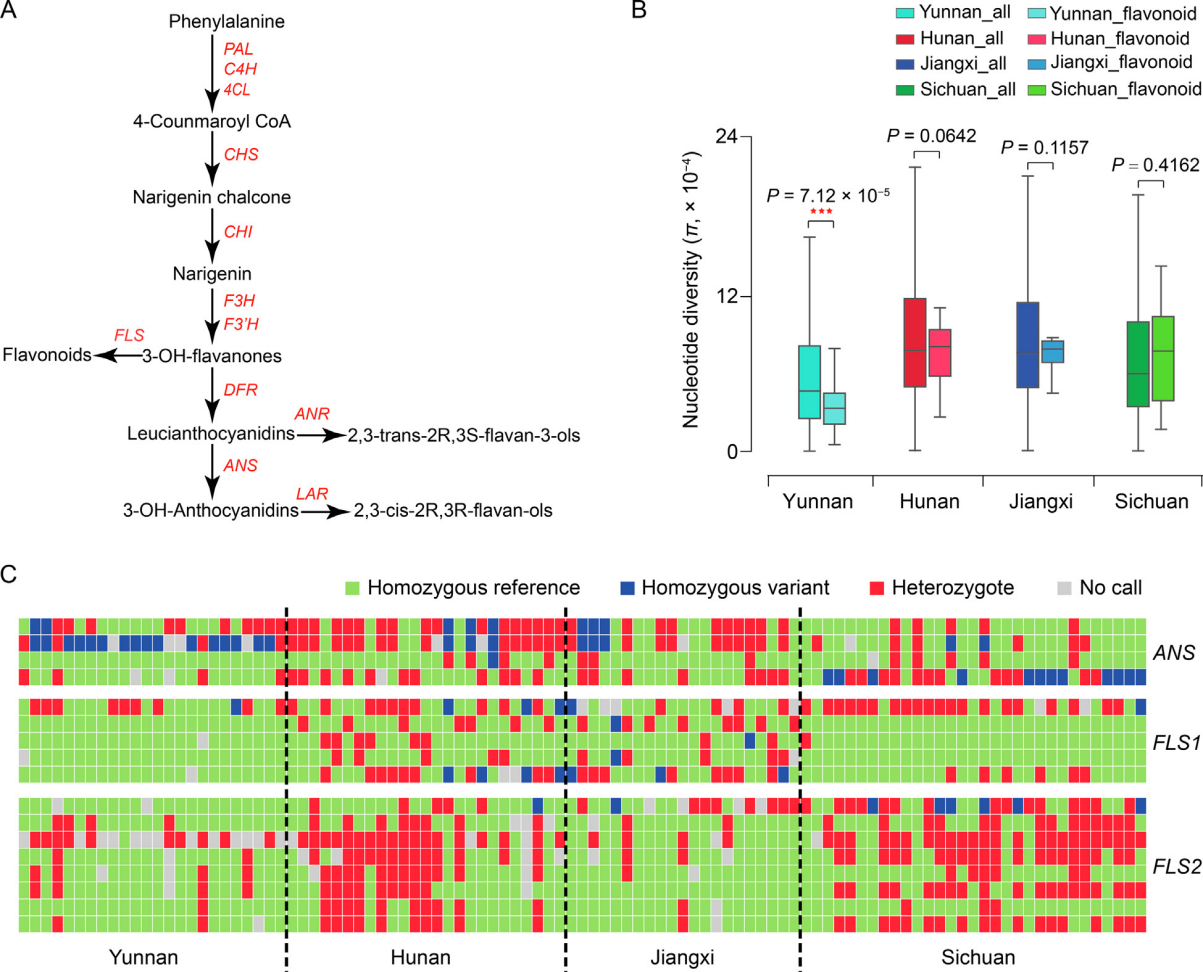
**Variations in flavonoid biosynthesis-related genes in the four groups of Shanmei** **A.** Schematic of the flavonoid biosynthesis pathway. **B.** Nucleotide diversity comparisons between flavonoid biosynthesis-related genes and all the genes in the genome of Shanmei for each of the four Shanmei groups. **C.** Genotype variations at non-synonymous SNPs in genes involved in the biosynthesis of flavonoids. *FLS1*, flavonol synthase copy 1; *FLS2*, flavonol synthase copy 2; *PAL*, phenylalanine ammonialyase; *C4H*, cinnamate 4-hydroxylase; *4CL*, cinnamate 4-hydroxylase; *CHS*, chalcone synthase; *CHI*, chalcone isomerase; *F3H*, flavanone 3-hydroxylase; *F3’H*, flavonoid 3'-hydroxylase; *DFR*, dihydroflavonol reductase; *ANS*, anthocyanidin synthase; *ANR*, anthocyanidin reductase; *LAR*, leucoanthocyanidin reductase.

**Supplementary Figure S12:**
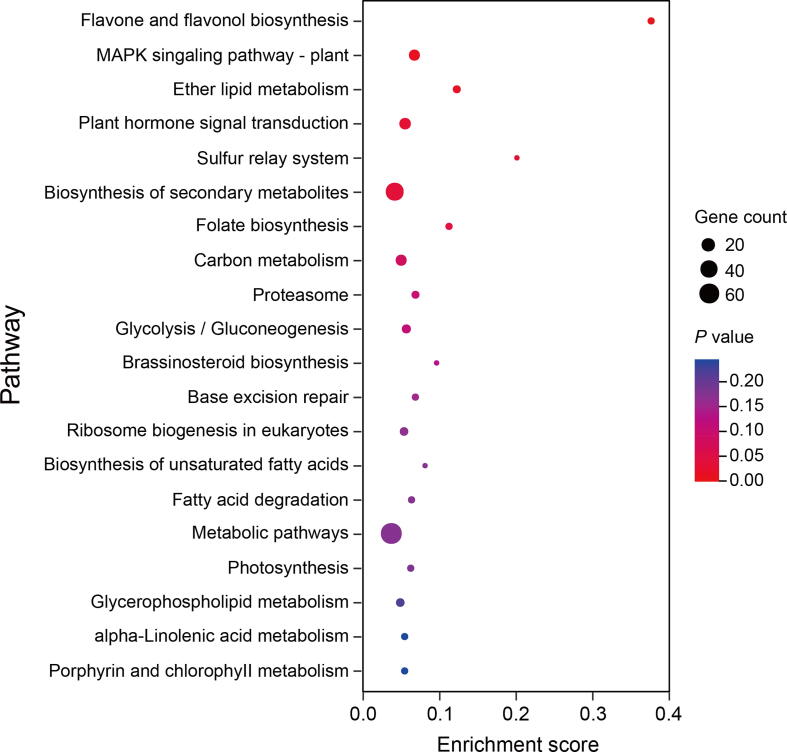
**Figure S12KEGG enrichment analysis of genes located at genomic regions under selection in the Shanmei Yunnan group compared to that of the Hunan group**

**Supplementary Figure S13:**
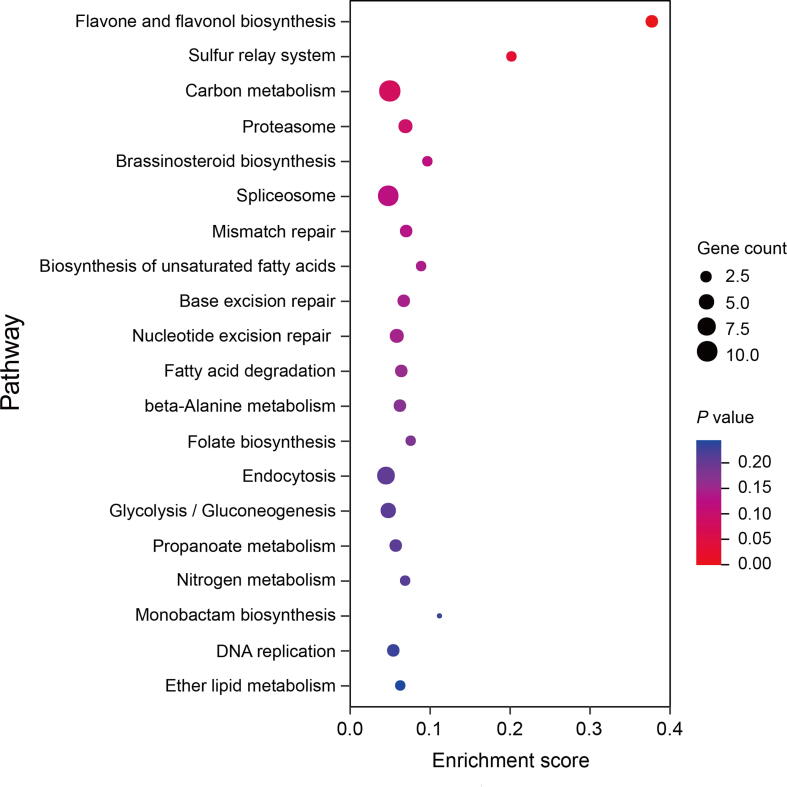
**Figure S13**  **KEGG enrichment analysis of genes located at genomic regions under selection in the Shanmei Yunnan group compared to that of the Jiangxi group**

**Supplementary Figure S14:**
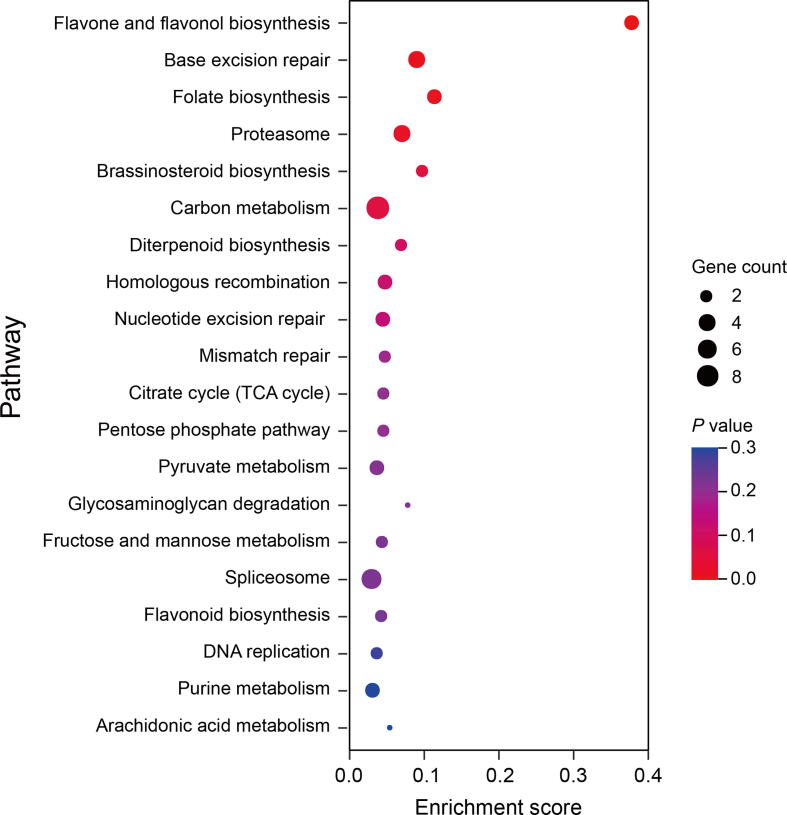
**Figure S14**  **KEGG enrichment analysis of genes located at genomic regions under selection in the Shanmei Yunnan group compared to that of the Sichuan group**

We also found that the flavonoid biosynthesis-related genes were strongly enriched in genes located at genomic regions under selection (Figure 4F, Figures S12, S13, and S14). By comparing the nucleotide diversity of genes involved in the flavonoid biosynthesis pathway (Figure 5A), we found that the Yunnan group had the lowest polymorphism (*π* = 4.07 × 10^−^^4^), indicating strong selection of these genes in the Yunnan group (Figure 5B). Considering that the genome-wide diversity of the Yunnan group is lower than that of the other groups, we further compared the diversity of flavonoid biosynthesis-related genes with that of all genes (used as the genome background). The results showed that the *π* values of flavonoid biosynthesis-related genes were significantly lower than those of the genome background in the Yunnan group, which were not significant in the other groups (Figure 5B). Moreover, *FLS1/2* encoding flavonol synthase and *ANS* encoding anthocyanidin synthase showed marked variations between the Yunnan group and the other groups (Figure 5C). *ANS* is a key component in anthocyanin biosynthesis, which is responsible for leaf or fruit coloring, as well as the responses of plants to changes in the external environment [33]. A high abundance of *ANS* enhances the resistance of bell pepper to low temperature and ultraviolet-B radiation [34]. Furthermore, *FLS* exhibits great potential for regulating plant growth and development, as well as enhancing plant resistance under abiotic stresses. For example, the increased expression of *CitFLS* promoted fruit ripening during citrus fruit development [35]. *FLS* also contributes to the acclimation of plants to salinity and ultraviolet-B [36]. The purifying selection of *FLS* and *ANS* in the Yunnan group indicates their contribution to the local environmental adaptability of Shanmei in Yunnan.

Additionally, some genes related to the mitogen-activated protein kinase (MAPK) signaling pathway and the plant hormone signal transduction pathway were also enriched in genes under selection. MAPK plays an important role in the plant response to stress [37]. In our study, *MKK2*, *ANP1*, and *MAPKKK17/18*, the key genes involved in the MAPK signaling pathway, were found to be under selection. Phytohormones play important roles in various biological activities of plants. They are the endogenous messenger molecules that precisely mediate plant growth and development as well as response to various biotic and abiotic stresses. Genes involved in the phytohormone signaling pathways were under selection in the Yunnan group, such as genes involved in abscisic acid (ABA) signaling (*PYL*, *PP2C*, and *NCED*) and auxin signaling (*IAA*, *ARF*, and *SAUR*). These results suggest the importance of the signaling pathways of MAPK and plant hormones in the environmental adaptability of Shanmei.

## Discussion

We assembled the first chromosome-scale genome of Shanmei and resequenced 101 Shanmei samples collected from different geographical regions of South China. We revealed that the genome of Shanmei had overall good synteny to the genomes of Fupenzi and blackberry, except for one large genomic translocation between Shanmei and blackberry, and four smaller genomic inversions between Shanmei and Fupenzi. We also found expanded gene families involved in flavonoid biosynthesis and stress resistance in the Shanmei genome, which contributed to its wide environmental adaptation. Furthermore, we generated a variome dataset of Shanmei, the analysis of which showed that the Hunan group of Shanmei was the more ancestral population. In contrast, the Yunnan group underwent strong selection, in which genes related to flavonoid biosynthesis and plant hormone signal transduction were subjected to natural selection. The assembled genome and the variome dataset generated in this study serve as valuable resources for future evolutionary and economic studies of Shanmei.

The genomic features of Shanmei are shaped by its adaptability to its wide habitat and ecological niche. Shanmei is a wild species with a broad distribution. The strong environmental adaptability and high barren tolerance of Shanmei make it a good pioneer plant for reclaiming wasteland. The assembled genome provides important information on the genetic bases underlying the adaptability of Shanmei. Interspecies comparative genomics analysis revealed that *HSPs*, *RPM1*, *BIN2*, and *BRI1* underwent significant gene copy number expansion in the Shanmei genome. The *HSP* genes have the function of enhancing the heat stress ability of plants [38], while the *RPM1* gene can reduce the damage caused by pathogens [26]. The expression of *BIN2* and *BRI1*, involved in brassinosteroid signal transduction, was found to be significantly increased under heat, salt, heavy metal, and drought stress [39], [40]. The expansion of these genes should contribute to its adaptation to broad geographical regions with varying environmental conditions. Shanmei is a typical low shrub that grows between the ground-covering grass and the arborescent stratum. We found that the copy numbers of the key genes related to lignin biosynthesis, such as *CAD* and *COMT*, increased generally in a gradient fashion in herbs, shrubs (Shanmei), and trees. These genes are associated with plant height. For example, the *CAD* and *CCR* mutations displayed a severe dwarfing phenotype [28], and the *COMT* and *CCoAOMT* double mutation resulted in reduced lignin and dwarfing in *Medicago truncatula* [41]. We speculated that the increase in the copy numbers of these genes may contribute to the increase in their expression dosages, which in turn is associated with the differences in the height phenotype, thus explaining the medium size of the Shanmei plants.

Selection for environmental adaptation-related genes was found in the Shanmei population, which is in contrast to that observed in domesticated crop species. Selective sweep analysis focusing on the high-altitude Yunnan group found that this group underwent stronger selection compared with the other groups. Flavonoid biosynthesis-related genes, as well as genes functioning in plant hormone signal transduction, were determined to be enriched in these genomic regions under selection. This indicates that these two sorts of pathways are crucial to the adaptation of the Yunnan group to its environment. Generally, flavonoids protect plants against ultraviolet light and high temperatures, as well as other stresses [42], [43]. It is therefore reasonable that flavonoid biosynthesis-related pathways were under selection in the high-altitude Yunnan group. Moreover, among these genes regulating flavonoid biosynthesis, we further found that *FLS* and *ANS* exhibited stronger selection in the Yunnan group. FLS and ANS function in catalyzing the biosynthesis of anthocyanins and flavanols, respectively. Thus, these two types of genes may contribute to the enhanced resistance of Shanmei to specific environmental factors such as the strong ultraviolet light in Yunnan [44], [45]. These findings on the selection of flavonoid biosynthesis-related genes were based on the population genomic data, and future studies with expression and metabolite data should further explore the molecular mechanisms underlying the adaptive evolution of Shanmei. Additionally, key genes related to ABA were identified as being under selection in Shanmei, including the genes *PYL*, *PP2C*, and *NCED*, which are essential for ABA biosynthesis during salt and drought stresses [46]. Taken together, these genes under selection are important for enhancing the environmental adaptability of Shanmei. Since Shanmei is a wild species, the pressure for survival and fitness in the wild environment is significant. These targeted genes in Shanmei are clearly different from those genes under selection in crop populations, in which genes regulating product-related traits, including yield and quality, are always found to be under strong selection.

In China, Shanmei is mainly distributed along a line from South to Northeast (Jilin Province) to Southwest (Tibet Autonomous Region) China, as indicated by the data provided by the Chinese Virtual Herbarium. Provinces with high Shanmei population density are located in South China, including Hunan, Jiangxi, Zhejiang, Sichuan, Guizhou, and Guangxi, as well as Chongqing Municipality. Based on this information, we collected Shanmei samples from four representative regions: the Hunan group, which is located in the central region of South China; the Jiangxi group, which is located in the east of South China; the Sichuan group, which is located in the west of South China; and the Yunnan group, which is located in the high-altitude region of Southeast China. The four Shanmei groups were served as an initial sample population to investigate the Shanmei population structure and evolutionary relationships, as well as to explore the specific sample groups under different environmental selection gradients. In the phylogenetic and genetic structure analyses, we found that the four groups of Shanmei were clearly separated into four clusters. The position of the outgroup and the genetic mixture data indicate that the Hunan group is the ancestral group for Shanmei. The clear clustering of the four phylogenetic groups suggests that the sampling of our Shanmei samples may not reflect the continuous evolutionary relationships of Shanmei from different geographical regions. We cannot rule out that our sampling was non-uniform, as Shanmei is widely distributed in large, wild regions. Furthermore, considering that we only sampled four geographical regions, there could be regions within the vicinity of the Hunan group where the most ancestral group of Shanmei is located. Nevertheless, this is the first study to reveal the general population structure of Shanmei, providing insight into the origin of this important species.

In summary, the high-quality chromosome-level genome and the variome dataset of Shanmei released here are valuable for investigating Shanmei and closely-related Rosaceae species. Our findings on the selection, population structure, and phylogenetic relationships will benefit the further research, material selection, and cultivation of Shanmei in the future.

## Materials and methods

### Materials, sampling, and sequencing

Shanmei seedlings were collected in Jiangxi Province in China (115.98°E, 29.68°N) and were transplanted into the greenhouse of the Chinese Academy of Agricultural Science. The genomic DNA was isolated from the tender leaves using the DNeasy plant mini kit (Catalog No. 69104, Qiagen, Dusseldorf, Germany). The Nanopore library was built according to the manufacturer’s protocol, and genome sequencing was performed to generate long reads using the Oxford Nanopore PromethION P48 sequencer (Biomarker, Beijing, China). For Illumina sequencing, a paired-end library was constructed with an insert size of 350 bp and sequenced using the Illumina HiSeq 4000 system (Biomarker), which was used to estimate genome and sequence characteristics. Details of the sequencing are provided in Table S1.

Considering that Shanmei is mainly distributed along a line from South to Northeast to Southwest China (https://www.cvh.ac.cn), samples from four representative regions were collected. These included the Hunan population (114.43°E, 27.29°N, 1000–1300 m) located in the central region of South China, the Jiangxi population (115.98°E, 29.68°N, 1100–1300 m) located in the east of South China, the Sichuan population (103.22°E, 29.35°N, 1400–1600 m) located in the west of South China, and the Yunnan population (104.43°E, 23.15°N, 1700–1900 m) located in Southeast China. The Yunnan Shanmei samples are distributed in the high-altitude region, serving as a subpopulation under specific environmental selection. Then, 2 μg of DNA per sample was extracted from the fresh leaves using a standard cetyl trimethylammonium bromide extraction protocol. Sequencing libraries were constructed using the TruSeq Nano DNA HT Sample Preparation Kit (Catalog No. FC-121-4003, Illumina, San Diego, CA) following the manufacturer’s instructions. These libraries were sequenced by the Illumina NovaSeq 6000 system (Biomarker), and 150-bp paired-end reads were generated with insert sizes around 350 bp.

### Genome assembly

Jellyfish (version 2.3.0) [47] was used to calculate the *k*-mer depth distribution with the Illumina short reads, and GenomeScope (version 1.0) [48] was used to estimate the genome size and heterozygosity. Then, NextDenovo (version 2.0, https://github.com/Nextomics/NextDenovo) was used to assemble the Nanopore reads into contigs. Racon (version 1.3.2) [49] and Pilon (version 1.2.3) [50] were then used to refine the original contigs with the Nanopore and Illumina reads, each of which was run for three rounds. Finally, Purge Haplotigs (version 1.2.3) [22] was used to remove heterozygous segments to generate the final contigs. BUSCO was used to assess the completeness of the genome with the embryophyta_odb10 database [51]. Default parameters were used if not specified.

### Hi-C library construction and scaffolding

Fresh leaves were collected for Hi-C sequencing. The *Hind*III restriction enzyme (Catalog No. R0104S, Illumina) was used during the library preparation procedure. The high-quality library was sequenced using the Illumina NovaSeq 6000 system (Biomarker). The Hi-C reads were filtered by removing adapter sequences and low-quality reads using Trimmomatic (version 0.39) [52]. The retained Hi-C reads were aligned to the contigs using Juicer (version 1.5, https://github.com/aidenlab/juicer) to obtain the interaction matrix. ALLHiC (version 0.9.8) [53] was used to group, order, and orientate the contigs. Finally, the linking results were manually curated to correct mis-joins and mis-assemblies based on the Hi-C heatmap using JuicerBox (version 1.11.08) [54].

### Repetitive element prediction

LTR_retriever (version 2.7) [55] and RepeatModeler (version 1.0.4) [56] were used to construct the *de novo* repeat libraries. Then, cd-hit software was used to merge the resultant libraries into a non-redundant repeat library (parameters: -c 0.8 -as 0.8 -M 0). Finally, RepeatMasker (version open-4.0.7) [56] was applied to identify and mask the repeat sequences in the Shanmei genome based on the library.

### Prediction and annotation of protein-coding genes

An integrated approach was applied to predict the protein-coding genes by merging the results from homology-based searches, mRNA-seq assisted prediction, and *ab initio* prediction. For annotation of homologs, genome sequences of eight species (grape, strawberry, blackberry, apple, peach, pear, apricot, and Chinese rose) were collected from the genome database for Rosaceae and were then aligned to the Shanmei genome to identify the homologous genes using Exonerate (version 2.4.7) [57]. The *ab initio* gene prediction of the Shanmei genome was performed using GeneMark (version 4.61_lic) [58] and Augustus (version 3.3.3) [59]. The RNA-seq data from three tissues (roots, stems, and leaves) were used for transcriptome prediction. Specifically, Hisat2 (version 2.2.1) [60] and Stringtie (version 2.1.4) [61] were used to map RNA-seq reads to the assembled genome and to assemble the alignments into transcripts, respectively. TransDecoder (version 5.5.0, https://github.com/TransDecoder/TransDecoder) was used to identify the potential coding regions in the resultant transcripts. The RNA-seq reads were *de novo* assembled into transcripts by Trinity (version 2.11.0) [62] using the genome-guided mode, and PASA (version 2.3.1) [63] was used for gene prediction from these transcripts. Finally, EVidenceModeler (version 1.1.1) [64] was used to integrate all gene prediction datasets to generate the final gene set of Shanmei. The predicted protein-coding genes were aligned to the KEGG databases and annotated using KEGG Automatic Annotation Server [65] with an E-value threshold of 1 × 10^−5^.

### Gene expansion and contraction

To identify homologous genes among Shanmei and other plants, the protein sequences of Shanmei were aligned to those of other species (grape, strawberry, blackberry, apple, peach, pear, and Chinese rose) using OrthoFinder (version 2.2.7) [66] with an E-value threshold of 1 × 10^−5^. The protein sequences of single-copy genes were aligned using MUSCLE (version 3.8.31) [67], and the phylogenetic tree was constructed using RAxML (version 8.2.10) [68] with the ML algorithm. CAFE (version 4.2.1) [23] was used to identify the expanded and contracted gene families for each species. Default parameters were used if not specified.

### SNP calling and filtering

The paired-end resequencing reads were filtered with Trimmomatic (version 0.38) [52]. BWA-MEM (version 0.7.17) [69] was used to align the reads of each sample to the assembled genome. Then, the sequence alignment files were sorted and indexed using SAMtools (version 1.6) [70]. The GATK (version 1.7.0) [32] was employed to identify variants. In order to obtain high-confidence variants, raw variants were filtered using VCFtools (version 0.1.16) [71]. The filtering criteria were as follows: 1) only SNPs with consensus quality (minQ) ≥ 30 and average SNP depth (minDP) ≥ 10 were retained; 2) the multiallelic sites were filtered out; 3) only SNPs with minor allele frequency ≥ 0.01 and minor allele count ≥ 3 were kept; and 4) SNPs were further filtered based on LD (with the parameter: --indep-pairwise 100-kb 1 0.5). Finally, 759,241 high-quality SNPs were retained for subsequent analyses. The SNP annotation was performed using ANNOVAR (version 2010Feb15) [72], and SNPs were categorized into intergenic, upstream, downstream, intron, and exon types based on their relative locations compared with the annotated genes. The SNPs located in exons were further separated into synonymous and non-synonymous SNPs.

### Phylogenetic analysis and population structure

PHYLIP (version 3.696, https://evolution.genetics.washington.edu/phylip.html) was employed to infer the phylogenies of the Shanmei population based on the neighbor-joining algorithm, and MEGA7 (version 7.0) [73] was used to visualize the phylogenetic tree. PCA of autosomal SNPs was performed using SNPRelate (version 1.28.0) [74]. Structure analysis was performed using ADMIXTURE (version 1.3.0) [75]. The *K* values were set from 2 to 7 to estimate the population structure (with the parameters: -geno 0.05 -maf 0.0037 -hwe 0.0001). Finally, the smallest cross-validation (CV) value appeared at *K* = 4 (Figure S11).

### Inference of the historical effective population size

PSMC (version 0.6.5-r67) [76] was used to estimate the historical effective population size based on the whole-genome resequencing data of the four Shanmei groups. The mutation rate was assumed as μ = 1.9 × 10^−9^ mutations per bp per generation, which was estimated by r8s (version 1.8.1) [77]. One generation was considered as one year. Finally, the script psmc_plot.pl from the PSMC package was used to visualize the results.

### Genome-wide selection signal scanning

To identify genomic regions under selection in the Yunnan Shanmei group compared to the other groups, *F*_ST_ and *π* were calculated using the VCFtools (version 0.1.16) [71] with a 50-kb nonoverlapping sliding window. Putative selection targets with the top 5% of log_2_ ratios for both *π* and *F*_ST_ were identified in the Yunnan group compared to each of the other groups. The genes from the genomic regions under selection were analyzed with in-house scripts.

### Identification of key genes involved in anthocyanin and lignin biosynthesis

The genes involved in anthocyanin and lignin biosynthesis reported in *Arabidopsis* were collected as references. The BLASTP and SynOrths (version 1.5) [78] tools were used to search the Shanmei genome for homologous genes with an E-value threshold of 1 × 10^−20^. Genes supported by both tools were extracted for subsequent analysis using an in-house script. The genes were further confirmed by functional domain prediction in PfamScan (version 1.5, https://www.ebi.ac.uk/Tools/pfa/pfamscan/). The gene *MYB10* was identified based on *RiMYB10* (GenBank: 161878916) from red raspberry (*Rubus idaeus*) using MUMmer (version 4.0.0) [79]. The phylogenetic tree was built using MEGA7 (version 7.0) [73] with the neighbor-joining algorithm.

## Data availability

The genome assembly data have been deposited in the Genome Warehouse (GWH) [80] at the National Genomics Data Center (NGDC), Beijing Institute of Genomics (BIG), Chinese Academy of Sciences (CAS) / China National Center for Bioinformation (CNCB) (GWH: GWHBDNY00000000), which are publicly accessible at https://ngdc.cncb.ac.cn/gwh. The resequencing data have been deposited in the Genome Sequence Archive (GSA) [81] at the NGDC, BIG, CAS / CNCB (GSA: CRA004566), which are publicly accessible at https://ngdc.cncb.ac.cn/gsa.

## CRediT author statement

**Yinqing Yang:** Formal analysis, Investigation, Writing - original draft. **Kang Zhang:** Investigation, Software, Writing - review & editing. **Ya Xiao:** Validation. **Lingkui Zhang:** Methodology. **Yile Huang:** Methodology. **Xing Li:** Investigation. **Shumin Chen:** Investigation. **Yansong Peng:** Resources. **Shuhua Yang:** Conceptualization, Resources. **Yongbo Liu:** Conceptualization, Resources. **Feng Cheng:** Conceptualization, Supervision, Writing - review & editing, Funding acquisition. All authors have read and approved the final manuscript.

## Competing interests

The authors have declared no competing interests.
