# Supplementary material for: Genome Assembly and Population Resequencing Reveal the Geographical Divergence of Shanmei (Rubus corchorifolius)
Source: Genomics Proteomics Bioinformatics. 2022 May 25;20(6):1106–18. doi: 10.1016/j.gpb.2022.05.003 (PMC10225494; doi:10.1016/j.gpb.2022.05.003)
Supplement: Supplementary Table S1 [file mmc1.doc]

**Table S1** **The statistics of sequencing data used for the Shanmei genome assembly**

| **Libraries** | **Insert (bp)** | **Raw data (Gb)** | **Clean data (Gb)** | **Mean read length (bp)** | **Sequence coverage (x)** |
| --- | --- | --- | --- | --- | --- |
| Illumina reads | 350 | 15.17 | 15.06 | 150 | 75.3 |
| ONT reads | 20 k | 40.21 | 36.87 | 23,999 | 184 |
| Hi-C reads | -- | 44.32 | 43.56 | 150 | 220 |

*Note*: ONT, Oxford Nanopore Technologies; Hi-C, high-throughput chromosome conformation capture.
