# Supplementary material for: Genome Assembly and Population Resequencing Reveal the Geographical Divergence of Shanmei (Rubus corchorifolius)
Source: Genomics Proteomics Bioinformatics. 2022 May 25;20(6):1106–18. doi: 10.1016/j.gpb.2022.05.003 (PMC10225494; doi:10.1016/j.gpb.2022.05.003)
Supplement: Supplementary Table S2 [file mmc2.doc]

**Table S2 The length and the number of contigs in each chromosome of Shanmei**

| **Chromosome ID** | **Contig number** | **Length (bp)** |
| --- | --- | --- |
| Rf01 | 16 | 26,961,307 |
| Rf02 | 15 | 295,197,94 |
| Rf03 | 21 | 34,210,402 |
| Rf04 | 14 | 27,555,748 |
| Rf05 | 20 | 31,428,983 |
| Rf06 | 16 | 36,678,181 |
| Rf07 | 15 | 27,931,591 |
