# Supplementary material for: Genome Assembly and Population Resequencing Reveal the Geographical Divergence of Shanmei (Rubus corchorifolius)
Source: Genomics Proteomics Bioinformatics. 2022 May 25;20(6):1106–18. doi: 10.1016/j.gpb.2022.05.003 (PMC10225494; doi:10.1016/j.gpb.2022.05.003)
Supplement: Supplementary Table S3 [file mmc3.doc]

**Table S**3 The statistics of genes in each prediction process

|  | **Gene set** | **Number** | **Average mRNA length** (bp) | **Average CDS length** (bp) | **Average exon per gene** |
| --- | --- | --- | --- | --- | --- |
| *De novo* | Augustus | 36,639 | 2527.05 | 290.3 | 3.62 |
|  | GeneMark | 26,451 | 3424.09 | 222.07 | 5.56 |
| Homolog | Exonerate | 26,049 | 2494.59 | 206.85 | 4.76 |
| RNA-Seq | - | 38,703 | 5164.35 | 233.36 | 7.13 |
| EVidenceModeler | - | 26,696 | 2818.22 | 239 | 4.97 |

*Note*: CDS, coding sequence.
