# Supplementary material for: Genome Assembly and Population Resequencing Reveal the Geographical Divergence of Shanmei (Rubus corchorifolius)
Source: Genomics Proteomics Bioinformatics. 2022 May 25;20(6):1106–18. doi: 10.1016/j.gpb.2022.05.003 (PMC10225494; doi:10.1016/j.gpb.2022.05.003)
Supplement: Supplementary Table S4 [file mmc4.doc]

**Table S4 The statistics of different groups of transposable elements in the genome of Shanmei**

|  | **Number** | **Lenth (bp)** | **Rate (%)** |
| --- | --- | --- | --- |
| DNA | 5237 | 2,304,897 | 1.07 |
| LINE1 | 5789 | 2,310,954 | 1.07 |
| LTR | 27,041 | 24,286,238 | 11.26 |
| Simple repeats | 63,459 | 3,721,107 | 1.73 |
| Low complexity | 10,388 | 1,032,729 | 0.48 |
| Unclassified | 131,478 | 44,106,065 | 20.45 |
| Total | 243,392 | 77,326,546 | 35.85 |

*Note*: LINE, long interspersed nuclear elements; LTR, long terminal repeat.
