# Supplementary material for: Genome Assembly and Population Resequencing Reveal the Geographical Divergence of Shanmei (Rubus corchorifolius)
Source: Genomics Proteomics Bioinformatics. 2022 May 25;20(6):1106–18. doi: 10.1016/j.gpb.2022.05.003 (PMC10225494; doi:10.1016/j.gpb.2022.05.003)
Supplement: Supplementary Table S5 [file mmc5.doc]

**Table S**5 The KEGG enrichment analysis of expanded genes in the genome of Shanmei

| **KEGG ID** | **Description** | **Gene number** | **Corrected *P* value** |
| --- | --- | --- | --- |
| ko00943 | Isoflavonoid biosynthesis | 9 | 8.96E-05 |
| ko00909 | Sesquiterpenoid and triterpenoid biosynthesis | 32 | 1.55E-04 |
| ko00400 | Phenylalanine, tyrosine and tryptophan biosynthesis | 27 | 4.13E-04 |
| ko04626 | Plant-pathogen interaction | 34 | 6.88E-04 |
| ko00360 | Phenylalanine metabolism | 29 | 1.17E-03 |
| ko00460 | Cyanoamino acid metabolism | 25 | 1.81E-03 |
| ko00944 | Flavone and flavonol biosynthesis | 7 | 2.25E-03 |
| ko02010 | ABC transporters | 18 | 7.64E-03 |
| ko00940 | Phenylpropanoid biosynthesis | 59 | 7.64E-03 |
| ko00130 | Ubiquinone and other terpenoid-quinone biosynthesis | 21 | 7.64E-03 |
| ko00905 | Brassinosteroid biosynthesis | 11 | 1.68E-02 |
| ko01110 | Biosynthesis of secondary metabolites | 290 | 1.72E-02 |
| ko00901 | Indole alkaloid biosynthesis | 6 | 3.84E-02 |

*Note*: KEGG, Kyoto Encyclopedia of Genes and Genomes.
