# Supplementary material for: Genome Assembly and Population Resequencing Reveal the Geographical Divergence of Shanmei (Rubus corchorifolius)
Source: Genomics Proteomics Bioinformatics. 2022 May 25;20(6):1106–18. doi: 10.1016/j.gpb.2022.05.003 (PMC10225494; doi:10.1016/j.gpb.2022.05.003)
Supplement: Supplementary Table S6 [file mmc6.doc]

**Table S6 The KEGG enrichment analysis of contracted genes in the genome of Shanmei**

| **KEGG ID** | **Description** | **Gene number** | **Corrected *P* value** |
| --- | --- | --- | --- |
| ko00902 | Monoterpenoid biosynthesis | 6 | 1.06E-05 |
| ko00592 | alpha-Linolenic acid metabolism | 9 | 1.38E-02 |
| ko00910 | Nitrogen metabolism | 6 | 2.62E-02 |
