# Supplementary material for: Genome Assembly and Population Resequencing Reveal the Geographical Divergence of Shanmei (Rubus corchorifolius)
Source: Genomics Proteomics Bioinformatics. 2022 May 25;20(6):1106–18. doi: 10.1016/j.gpb.2022.05.003 (PMC10225494; doi:10.1016/j.gpb.2022.05.003)
Supplement: Supplementary Table S8 [file mmc8.doc]

**Table S8 Copy number variation of key genes for lignin biosynthesis in Rosaceae**

| **Gene name** | ***Fragaria vesca*** | ***Rosa chinensis*** | ***Rubus corchorifolius*** | ***Rubus occidentalis*** | ***Prunus armeniaca*** | ***Prunus mume*** | ***Prunus persica*** | ***Pyrus communis*** | ***Malus domestica*** | ***P*** value |
| --- | --- | --- | --- | --- | --- | --- | --- | --- | --- | --- |
| *PAL* | 2 | 2 | 2 | 2 | 2 | 2 | 2 | 4 | 4 | 0.995 |
| *C4H* | 2 | 2 | 2 | 2 | 2 | 2 | 2 | 4 | 4 | 0.995 |
| *4CL* | 6 | 6 | 6 | 5 | 5 | 6 | 6 | 6 | 6 | 0.965 |
| *CCR* | 1 | 1 | 1 | 1 | 2 | 3 | 3 | 3 | 2 | 0.673 |
| *CAD* | 12 | 14 | 9 | 11 | 18 | 19 | 20 | 22 | 20 | 0.036 |
| *C3H* | 1 | 2 | 1 | 1 | 3 | 3 | 4 | 4 | 4 | 0.626 |
| *COMT* | 5 | 11 | 7 | 6 | 12 | 14 | 14 | 15 | 14 | 0.047 |
| *CCoAOMT* | 5 | 6 | 7 | 8 | 7 | 6 | 7 | 9 | 9 | 0.947 |
| *F5H* | 2 | 2 | 2 | 2 | 2 | 2 | 2 | 4 | 4 | 0.995 |
