# Supplementary material for: Genome Assembly and Population Resequencing Reveal the Geographical Divergence of Shanmei (Rubus corchorifolius)
Source: Genomics Proteomics Bioinformatics. 2022 May 25;20(6):1106–18. doi: 10.1016/j.gpb.2022.05.003 (PMC10225494; doi:10.1016/j.gpb.2022.05.003)
Supplement: Supplementary Table S9 [file mmc9.doc]

**Table S9 Identification of key genes in anthocyanin biosynthesis**

| **Gene name** | **Ath id** | **NCBI** | **Ro id** | **Rf ID** | **Identify** |
| --- | --- | --- | --- | --- | --- |
| *PAL* | *AT2G37040.1* | *-* | *Ro06_G27621* | *Rf060219950.1* | 99.042 |
| *PAL* | *AT2G37040.1* | *-* | *Ro07_G16482* | *Rf070255320.1* | 98.243 |
| *4CL* | *AT1G51680.1* | *-* | *Ro04_G26593* | *Rf040115930.1* | 98.532 |
| *4CL* | *AT1G51680.1* | *-* | *Ro06_G14802* | *Rf060207250.1* | 97.794 |
| *4CL* | *AT1G51680.1* | *-* | *Ro07_G08962* | *Rf070266120.1* | 96.965 |
| *C4H* | *AT2G30490.1* | *-* | *Ro03_G10602* | *Rf030075160.1* | 91.036 |
| *C4H* | *AT2G30490.1* | *-* | *Ro04_G12638* | *Rf040118610.1* | 85.009 |
| *CHS* | *AT5G13930.1* | *-* | *Ro02_G01512* | *Rf020060010.1* | 94.832 |
| *CHS* | *AT5G13930.1* | *NO.JN602374* | *Ro04_G24959* | *Rf040123450.1* | 96.907 |
| *F3H* | *AT3G51240.1* | *NO.FJ554630* | *Ro01_G01174* | *Rf010022180.1* | 98.907 |
| *DFR* | *AT5G42800.1* | *NO.JF764809* | *Ro02_G02344* | *Rf020069420.1* | 92.063 |
| *ANS* | *AT4G22880.1* | *NO.JF764807* | *Ro05_G14884* | *Rf050170540.1* | 93.516 |
| *ANR* | *-* | *NO.JQ068825* | *Ro03_G10520* | *Rf030110430.1* | 97.899 |
| *LAR* | *-* | *NO.JQ068826* | *Ro04_G02628* | *Rf040139840.1* | 95.455 |
| *GT1* | *-* | *NO.JF764808* | *Ro04_G36884* | *Rf040121080.1* | 96.643 |
| *CHI* | *AT3G55120.1* | *NO.AY040321* | *Ro07_G11984* | *Rf070256510.1* | 96.218 |
| *MYB10* | *-* | *NO.JQ359611* | *Ro01_G29886* | *Rf010012550.1* | 71.053 |
| *FLS* | *AT5G08640.1* | *NO.AB038247* | *Ro02_G01409* | *Rf020058780.1* | 97.015 |
| *FLS* | *AT5G08640.1* | *-* | *Ro02_G01412* | *Rf020058800.1* | 93.976 |

*Note*: NCBI, National Center of Biotechnology Information; Rf, Shanmei; Ro, blackberry; Ath, *Arabidopsis*.
