# Supplementary material for: Genome Assembly and Population Resequencing Reveal the Geographical Divergence of Shanmei (Rubus corchorifolius)
Source: Genomics Proteomics Bioinformatics. 2022 May 25;20(6):1106–18. doi: 10.1016/j.gpb.2022.05.003 (PMC10225494; doi:10.1016/j.gpb.2022.05.003)
Supplement: Supplementary Table S11 [file mmc11.doc]

**Table S11 Distribution of SNPs in each chromosome of Shanmei**

| **Chromosome** | **SNP** | **Intergenic** | **Intron** | **Exon** | **Upstream** | **DownStream** | **Synonymous** | **Nonsynonymous** |
| --- | --- | --- | --- | --- | --- | --- | --- | --- |
| Rf01 | 96,808 | 57,402 | 14,681 | 9637 | 8532 | 7657 | 4972 | 4665 |
| Rf02 | 100,993 | 56,323 | 16,612 | 10,310 | 9931 | 9149 | 5466 | 4844 |
| Rf03 | 117,838 | 63,517 | 19,942 | 12,810 | 12,360 | 10,653 | 6593 | 6217 |
| Rf04 | 94,412 | 52,545 | 16,015 | 9550 | 9071 | 8352 | 5054 | 4496 |
| Rf05 | 110,339 | 63,427 | 17,379 | 10,861 | 10,570 | 9340 | 5666 | 5195 |
| Rf06 | 129,085 | 65,562 | 22,075 | 15,143 | 15,453 | 12,984 | 7871 | 7272 |
| Rf07 | 109,503 | 61,017 | 17,921 | 11,808 | 10,344 | 9638 | 6029 | 5779 |
| Total | 758,978 | 419,793 | 124,625 | 80,119 | 76,261 | 67,773 | 41,651 | 38,468 |

*Note*: SNP, single nucleotide polymorphism.
